# Mendelian randomization highlights significant difference and genetic heterogeneity in clinically diagnosed Alzheimer’s disease GWAS and self-report proxy phenotype GWAX

**DOI:** 10.1186/s13195-022-00963-3

**Published:** 2022-01-28

**Authors:** Haijie Liu, Yang Hu, Yan Zhang, Haihua Zhang, Shan Gao, Longcai Wang, Tao Wang, Zhifa Han, Bao-liang Sun, Guiyou Liu

**Affiliations:** 1 Department of Neurology, Xuanwu Hospital, Capital Medical University, Beijing, 100053 China; 2 School of Life Science and Technology, Harbin Institute of Technology, Harbin, 150080 China; 3 Department of Pathology, The Affiliated Hospital of Weifang Medical University, Weifang, 261053 China; 4 Beijing Institute of Brain Disorders, Laboratory of Brain Disorders, Ministry of Science and Technology, Collaborative Innovation Center for Brain Disorders, Capital Medical University, Beijing, 100069 China; 5 Department of Anesthesiology, The Affiliated Hospital of Weifang Medical University, Weifang, 261053 China; 6 Chinese Institute for Brain Research, Beijing, China; 7 State Key Laboratory of Medical Molecular Biology, Institute of Basic Medical Sciences, Chinese Academy of Medical Sciences, Beijing, China; 8 Key Laboratory of Cerebral Microcirculation in Universities of Shandong, Department of Neurology, Second Affiliated Hospital, Shandong First Medical University & Shandong Academy of Medical Sciences, Taian, 271000 Shandong China; 9 Beijing Key Laboratory of Hypoxia Translational Medicine, National Engineering Laboratory of Internet Medical Diagnosis and Treatment Technology, Xuanwu Hospital, Capital Medical University, Beijing, 100053 China

**Keywords:** Alzheimer’s disease, Mendelian randomization, GWAS, GWAX, Genetic heterogeneity

## Abstract

**Background:**

Until now, Mendelian randomization (MR) studies have investigated the causal association of risk factors with Alzheimer’s disease (AD) using large-scale AD genome-wide association studies (GWAS), GWAS by proxy (GWAX), and meta-analyses of GWAS and GWAX (GWAS+GWAX) datasets. However, it currently remains unclear about the consistency of MR estimates across these GWAS, GWAX, and GWAS+GWAX datasets.

**Methods:**

Here, we first selected 162 independent educational attainment genetic variants as the potential instrumental variables (*N* = 405,072). We then selected one AD GWAS dataset (*N* = 63,926), two AD GWAX datasets (*N* = 314,278 and 408,942), and three GWAS+GWAX datasets (*N* = 388,324, 455,258, and 472,868). Finally, we conducted a MR analysis to evaluate the impact of educational attainment on AD risk across these datasets. Meanwhile, we tested the genetic heterogeneity of educational attainment genetic variants across these datasets.

**Results:**

In AD GWAS dataset, MR analysis showed that each SD increase in years of schooling (about 3.6 years) was significantly associated with 29% reduced AD risk (OR=0.71, 95% CI: 0.60–0.84, and *P*=1.02E−04). In AD GWAX dataset, MR analysis highlighted that each SD increase in years of schooling significantly increased 84% AD risk (OR=1.84, 95% CI: 1.59–2.13, and *P*=4.66E−16). Meanwhile, MR analysis suggested the ambiguous findings in AD GWAS+GWAX datasets. Heterogeneity test indicated evidence of genetic heterogeneity in AD GWAS and GWAX datasets.

**Conclusions:**

We highlighted significant difference and genetic heterogeneity in clinically diagnosed AD GWAS and self-report proxy phenotype GWAX. Our MR findings are consistent with recent findings in AD genetic variants. Hence, the GWAX and GWAS+GWAX findings and MR findings from GWAX and GWAS+GWAX should be carefully interpreted and warrant further investigation using the AD GWAS dataset.

**Supplementary Information:**

The online version contains supplementary material available at 10.1186/s13195-022-00963-3.

## Background

Alzheimer’s disease (AD) is the most common neurodegenerative disease [1, 2]. From 2009 to 2019, large-scale genome-wide association studies (GWAS) have been conducted using clinically diagnosed AD and cognitively normal controls, and successfully identified multiple common AD genetic variants with genome-wide significance *P* < 5.00E−08 [3–9], especially two large-scale GWAS meta-analyses from the International Genomics of Alzheimer’s Project (IGAP) including the IGAP 2013 (*n*= 74,046, 25,580 cases, and 48,466 controls) [6], and the IGAP 2019 (*n*= 94,437, including 35,274 cases and 59,163 controls) [9]. However, these genetic variants could only explain about 40% of the genetic variance of AD [10–12]. Hence, the majority of AD genetic risk remains undiscovered [10–12]. Until recently, GWAS for family history of AD, known as GWAS by AD proxy phenotype (GWAX) using UK Biobank individuals is widely used to increase the sample size into the traditional GWAS, which may contribute to identify more additional novel genetic variants [13–15]. These meta-analyses of AD GWAS and GWAX (GWAS+GWAX) have reported novel findings [13–15].

Importantly, all these AD GWAS, GWAX, and GWAS+GWAX summary datasets are publicly available, which provides strong data support to investigate the causal association between AD and previously reported risk factors using a Mendelian randomization (MR) design [16–18]. Meanwhile, there is no strict standard to limit the use of AD GWAS, GWAX, or GWAS+GWAX datasets in MR studies. Hence, some MR studies are based on the AD GWAS datasets from IGAP 2013 or IGAP 2019 [19–25], of which three MR studies had evaluated the impact of multiple modifiable risk factors on AD [16–18]. In 2017, Larsson and colleagues selected 24 potentially modifiable risk factors and found that only increased educational attainment was significantly associated with a reduced risk of AD [16]. In 2020, Wang and colleagues analyzed 45 potentially modifiable risk factors, and eventually highlighted educational attainment and urate levels [17]. In 2021, Andrews and colleagues selected 22 risk factors, and eventually identified educational attainment using polygenic risk scores (PRS) and MR [18]. Meanwhile, others MR studies are based on the AD GWAS+GWAX datasets [26–32]. However, it currently remains unclear about the consistency of MR estimates from AD GWAS, GWAX, and GWAS+GWAX datasets.

Here, we selected the educational attainment as the risk factor, and evaluated its impact on AD risk using large-scale AD GWAS, GWAX, or GWAS+GWAX datasets, as the causal association between educational attainment and AD had been well established in recent MR studies [16–18, 33].

## Methods

### Study design

**Fig. 1:**
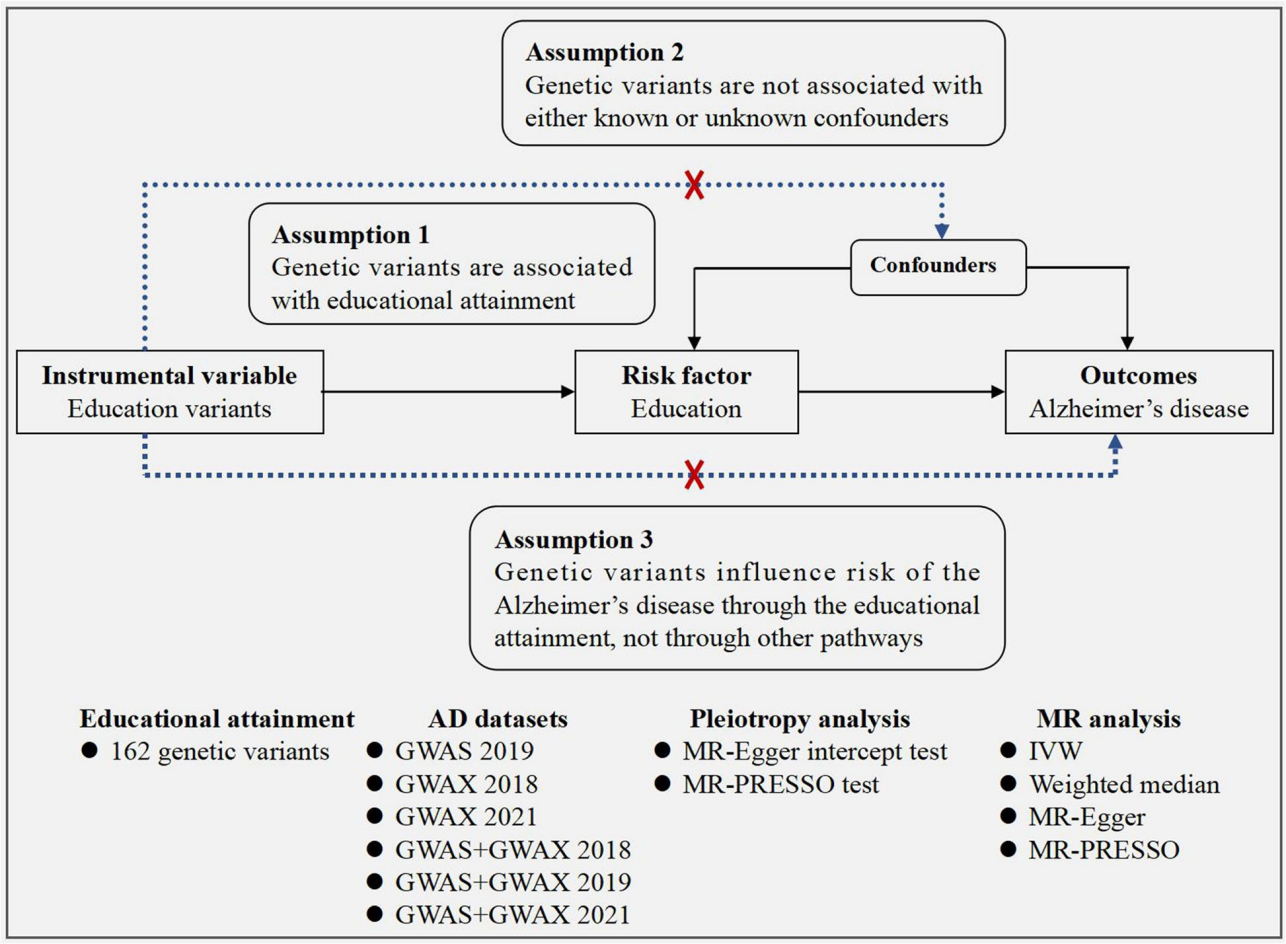
The flow chart about the MR study design. GWAS, genome-wide association studies; GWAX, GWAS by proxy; GWAS+GWAX, meta-analyses of GWAS and GWAX; IVW, Inverse-variance weighted; MR-PRESSO, Mendelian randomization pleiotropy residual sum and outlier

MR is based on three principal assumptions. First, the instrumental variables (genetic variants) should be significantly associated with the exposure (educational attainment), generally achieving the genome-wide significant level (*P*<5.00E-08) [19]. Second, instrumental variables should not be associated with confounders of the exposure (educational attainment) and outcome (AD) [19]. Third, instrumental variables should affect the risk of the outcome (AD) only through exposure (educational attainment) [19]. The second and third assumptions are collectively known as independence from pleiotropy. Figure 1 provides a flow chart about our MR study design.

### Educational attainment genetic variants

We selected 162 independent educational attainment genetic variants with the genome-wide significance (*P* < 5.00E−08) to be the potential instrumental variables, as provided in supplementary Table 1 [34]. Educational attainment is a continuous variable, which is determined by the number of years of schooling completed at or above age 30 [34]. These 162 genetic variants are identified by a large-scale GWAS in 405,072 individuals of European descent including 293,723 individuals in discovery stage (SSGAC) and 111,349 individuals in the independent replication stage (UK Biobank) [34].

### AD GWAS dataset

We selected the clinically diagnosed AD GWAS dataset from IGAP 2019 stage 1 including 21,982 AD cases and 41,944 cognitively normal controls of European descent [9]. This GWAS dataset is based on a meta-analysis using participants from four AD consortia including Alzheimer Disease Genetics Consortium, Cohorts for Heart and Aging Research in Genomic Epidemiology Consortium (CHARGE), The European Alzheimer’s Disease Initiative (EADI), and Genetic and Environmental Risk in AD/Defining Genetic, Polygenic and Environmental Risk for Alzheimer’s Disease Consortium (GERAD/PERADES) [9]. Table 1 provides the demographic profile about the AD GWAS dataset.

**Table 1:** Demographic profile about the selected AD GWAS datasets

Dataset	AD			Control		
	*N*	% female	Mean AAO (s.d)	*N*	% female	Mean AAE (s.d)
GWAS ADGC [9]	14,428	59.3	71.1 (17.3)	14,562	59.3	76.2 (9.9)
GWAS CHARGE [9]	2,137	67.3	82.6 (12)	13,474	55.8	76.7 (8.2)
GWAS EADI [9]	2,240	65	75.4 (9.1)	6631	60.6	78.9 (7.0)
GWAS GERAD [9]	3,177	64	73.0 (0.2)	7277	51.8	51.0 (0.1)
GWAS All [9]	21,982	-	-	41,944	-	-
GWAX 2018 [13]	42,034	65.9	-	272,244	-	-
GWAX 2021 [15]	53,042^a^	-	-	355,900	-	-
GWAS+GWAX 2018 [13]	67,614	-	-	320,710	-	-
GWAS+GWAX 2019 [14]	71,880	-	-	383,378	-	-
GWAS+GWAX 2021 [15]	75,024	-	-	397,844	-	-

*AD* Alzheimer’s disease, *AAO* age at onset, *AAE* age at examination, *s.d* standard deviation, *GWAS* genome-wide association studies, *GWAX* GWAS by proxy, *GWAS+GWAX* meta-analyses of GWAS and GWAX

^a^These 53,042 AD cases consisted of 898 clinically diagnosed AD and 52,791 AD proxy phenotype

### AD GWAX datasets

We selected two GWAX datasets for AD proxy phenotype including GWAX 2018 [13], and GWAX 2021 [15], respectively. Both studies are based on the UK Biobank participants [35]. UK Biobank is a large national and international health resource including the genetic and phenotype data from 502,536 community-dwelling individuals aged between 37 and 73 years recruited in the United Kingdom between 2006 and 2010 [35]. In UK Biobank, a proxy phenotype for AD case-control status was assessed via self-report [13]. Participants were asked to report “Has/did your father or mother ever suffer from Alzheimer’s disease/dementia?” [13]. Table 1 provides the demographic profile about these two AD GWAX datasets.

### AD GWAS+GWAX datasets

We selected three large-scale AD GWAS+GWAX datasets including GWAS+GWAX 2018 (a meta-analysis using IGAP 2013 and UK Biobank) [13], GWAS+GWAX 2019 (a meta-analysis using IGAP 2013, PGC-ALZ, ADSP, and UK Biobank) [14], and GWAS+GWAX 2021 (a meta-analysis using IGAP 2019 and UK Biobank) [15], respectively. All these three GWAS+GWAX datasets included the IGAP and UK Biobank participants, and are not independent of each other. Table 1 provides the demographic profile about the three GWAS+GWAX datasets.

### Establishing the Wald estimator

For the same effect allele from each educational attainment genetic variant *G*_*j*_(*j* = 1, …, 162), we assume that we have successfully extracted their corresponding summary results in educational attainment GWAS dataset including the beta coefficients and their standard errors (${\hat{\beta}}_{Xj}, se\left({\hat{\beta}}_{Xj}\right)$), and in the AD GWAS, GWAX and GWAS+GWAX datasets including the beta coefficients and their standard errors (${\hat{\beta}}_{Yj}, se\left({\hat{\beta}}_{Yj}\right)$). For a given genetic variant, the causal effect of educational attainment on AD can be consistently estimated as a simple ratio, also called the Wald estimator ${\hat{\theta}}_j=\frac{{\hat{\beta}}_{Yj}}{{\hat{\beta}}_{Xj}}$ and its approximate variance ${v}_j=\frac{se{\left({\hat{\beta}}_{Yj}\right)}^2}{{{\hat{\beta}}_{Xj}}^2}$ [19, 36].

### MR analysis

Using the Wald estimator from each educational attainment genetic variant, we conducted the MR analysis using four MR methods including inverse-variance weighted (IVW), weighted median, MR-Egger, and MR-PRESSO (Mendelian Randomization Pleiotropy RESidual Sum and Outlier) to combine the variant-specific estimates and get the overall estimate [36–39]. IVW is the main MR analysis method, which combines the variant-specific Wald estimators by taking the inverse of their approximate variances as the corresponding weights [37]. Weighted median could derive consistent estimates when up to 50% of instruments are not valid [37]. MR-Egger could test the presence of potential pleiotropy and account for this potential pleiotropy using the MR-Egger intercept test [36]. MR-PRESSO could detect and correct for the horizontal pleiotropy via outlier removal (the MR-PRESSO outlier test) [40]. The odds ratio (OR) as well as 95% confidence interval (CI) of AD corresponds to about per 3.6 years increase (about 1 standard deviation (SD)) in EduYears. All the statistical tests were completed using R Packages “MendelianRandomization” [39] and “MR-PRESSO” [40]. The significance threshold is *P* < 0.05.

### Heterogeneity test and paired-samples *T* test

We performed a heterogeneity test of the Wald estimators using the Cochran’s Q statistic and the *I*^2^ statistic [41]. Cochran’s Q statistic approximately follows a *χ*^2^ distribution with k-1 degrees of freedom (k is the number of the selected studies) [42]. ${I}^2=\left(\mathrm{Q}-\left(\mathrm{k}-1\right)\right)\left/ Q\times 100\%\right.$ ranges from 0 to 100%, with 0–25%, 25–50%, 50–75%, and 75–100% corresponding to low, moderate, large and extreme heterogeneity, respectively [42]. Importantly, the Cochran’s Q statistic and *I*^2^ assume that the subjects are independent of one another and were selected at random from a larger population. Hence, we only test the heterogeneity in AD GWAS and GWAX including two comparisons GWAS vs. GWAX 2018, and GWAS vs. GWAX 2021. All statistical tests were completed using R Package “meta: General Package for Meta-Analysis.”

In line with the heterogeneity test, we further conducted the paired-samples *T* test to evaluate the average differences of Wald estimators in GWAS vs. GWAX 2018, and GWAS vs. GWAX 2021. Analysis of variance (ANOVA) is widely used to analyze the differences among means from multiple independent (unrelated) groups. However, the selected AD GWAS, GWAX, and GWAS+GWAX datasets are not completely independent with each other. Therefore we could not provide the overall differences among the mean effect sizes across the AD GWAS, GWAX, and GWAS+GWAX datasets. Here, we provide a combined plot using all shared genetic variants to visualize the differences in effect sizes and directions across the six datasets.

## Results

### MR analysis in AD GWAS dataset

We extracted the GWAS summary statistics of 159 educational attainment genetic variants in the AD GWAS dataset, as provided in supplementary Table 2. Using the MR-Egger intercept test, we did not identify any significant pleiotropic variant. Using MR-PRESSO Global Test, we found two genetic variants rs268134 and rs28420834 to be the pleiotropic variants (Table 2). Hence, we excluded both variants in MR analysis. IVW showed that each SD increase in years of schooling (about 3.6 years) was significantly associated with 29% reduced AD risk (OR=0.71, 95% CI: 0.60–0.84, and *P*=1.02E−04). Interestingly, evidence from weighted median, MR-Egger, and MR-PRESSO further supported this finding, as provided in Table 3. Meanwhile, all the MR estimates from these four methods are consistent in terms of direction.

**Table 2:** Pleiotropy analysis in AD GWAS, GWAX, and GWAS+GWAX datasets

GWAS dataset	SNP #	MR-Egger intercept			MR-PRESSO	
		Intercept	95% CI	*P* value	*P* value	Pleiotropy variant
GWAS	159	0.01	[− 0.002, 0.023]	0.108	0.0025	rs268134, rs28420834
GWAX 2018	147	0.003	[− 0.002, 0.008]	0.189	0.085	No significant outliers
GWAX 2021	159	− 0.001	[− 0.012, 0.009]	0.809	0.02925	rs268134
GWAS+GWAX 2018	147	0.001	[− 0.004, 0.006]	0.725	0.01375	No significant outliers
GWAS+GWAX 2019	155	0.001	[− 0.001, 0.003]	0.226	0.06875	No significant outliers
GWAS+GWAX 2021	159	0.003	[− 0.006, 0.011]	0.536	0.001	rs268134

The significance threshold is *P* < 0.05

*GWAS* genome-wide association studies, *GWAX* GWAS by proxy, *GWAS+GWAX* meta-analyses of GWAS and GWAX, *MR-PRESSO* Mendelian randomization pleiotropy residual sum and outlier

**Table 3:** MR analysis of the association between educational attainment and AD

Dataset	Method	OR	95% CI	*P* value
GWAS	IVW	0.71	0.60–0.84	1.02E−04
	Weighted median	0.69	0.54–0.88	2.00E−03
	MR-Egger	0.39	0.19–0.80	1.00E−02
	MR-PRESSO	0.71	0.60–0.84	1.40E−04
GWAX 2018	IVW	1.09	1.00–1.19	5.10E−02
	Weighted median	1.01	0.89–1.16	8.62E−01
	MR-Egger	0.93	0.72–1.20	5.83E−01
	MR-PRESSO	1.09	1.00–1.20	5.26E−02
GWAX 2021	IVW	1.84	1.59–2.13	4.66E−16
	Weighted median	1.88	1.54–2.30	1.23E−09
	MR-Egger	2.10	1.16–3.79	1.40E−02
	MR-PRESSO	1.84	1.59–2.13	1.22E−13
GWAS+GWAX 2018	IVW	1.00	0.91–1.08	9.10E−01
	Weighted median	0.92	0.82–1.04	1.70E−01
	MR-Egger	0.95	0.74–1.23	7.12E−01
	MR-PRESSO	1.00	0.91–1.08	9.10E−01
GWAS+GWAX 2019	IVW	0.96	0.93–0.98	2.00E−03
	Weighted median	0.95	0.92–0.99	1.00E−02
	MR-Egger	0.90	0.80–1.00	5.20E−02
	MR-PRESSO	0.96	0.93–0.98	1.82E−03
GWAS+GWAX 2021	IVW	1.22	1.08–1.36	1.00E−03
	Weighted median	1.19	1.02–1.39	3.00E−02
	MR-Egger	1.12	0.70–1.79	6.44E−01
	MR-PRESSO	1.22	1.08–1.36	1.11E−03

The significance of the association between educational attainment and AD was at *P* < 0.05

*CI* confidence interval, *IVW* inverse-variance weighted, *MR-PRESSO* Mendelian randomization pleiotropy residual sum and outlier, *GWAS* genome-wide association studies, *GWAX* GWAS by proxy, *GWAS+GWAX* meta-analyses of GWAS and GWAX

### MR analysis in AD GWAX datasets

We extracted the GWAS summary statistics of 147 and 159 educational attainment genetic variants in GWAX 2018, and GWAX 2021, respectively, as provided in supplementary Table 3-4. In GWAX 2018, no pleiotropic variant is identified using both the MR-Egger intercept test and MR-PRESSO Global Test (Table 2). MR analysis indicated no significant causal association between educational attainment and AD, as provided in Table 3. However, two methods showed an increased trend of AD risk with high educational attainment including IVW (OR=1.09, 95% CI: 1.00–1.19, and *P*=0.051), and MR-PRESSO (OR=1.09, 95% CI: 1.00–1.20, and *P*=0.053). In GWAX 2021, rs268134 is identified to be a pleiotropic variant and then excluded in MR analysis (Table 2). IVW highlighted that each SD increase in years of schooling could significantly increase 84% AD risk (OR=1.84, 95% CI: 1.59–2.13, and *P*=4.66E−16). The MR estimates from weighted median, MR-Egger and MR-PRESSO were consistent with the IVW estimate in terms of direction and magnitude (Table 3).

### MR analysis in AD GWAS+GWAX datasets

We extracted the GWAS summary statistics of 147, 155, and 159 educational attainment genetic variants in GWAS+GWAX 2018, GWAS+GWAX 2019, and GWAS+GWAX 2021, respectively, as provided in supplementary Table 5-7. Only in GWAS+GWAX 2021, MR-PRESSO indicated rs268134 to be a pleiotropic variant, and then excluded in MR analysis (Table 2). In GWAS+GWAX 2018, MR analysis showed no significant causal association between educational attainment and AD (Table 3). In GWAS+GWAX 2019, we found that high educational attainment could reduce the risk of AD (Table 3). In GWAS+GWAX 2021, MR analysis further supported the increased risk of AD with high educational attainment using four MR methods, as provided in Table 3. IVW showed that each SD increase in years of schooling could significantly increase 22% AD risk (OR=1.22, 95% CI: 1.08–1.36, and *P*=1.00E−03). The MR estimates from weighted median, MR-Egger and MR-PRESSO were consistent with the IVW estimate in terms of direction and magnitude (Table 3).

### MR analysis in AD GWAS, GWAX, and GWAS+GWAX datasets using the same genetic variants

**Fig. 2:**
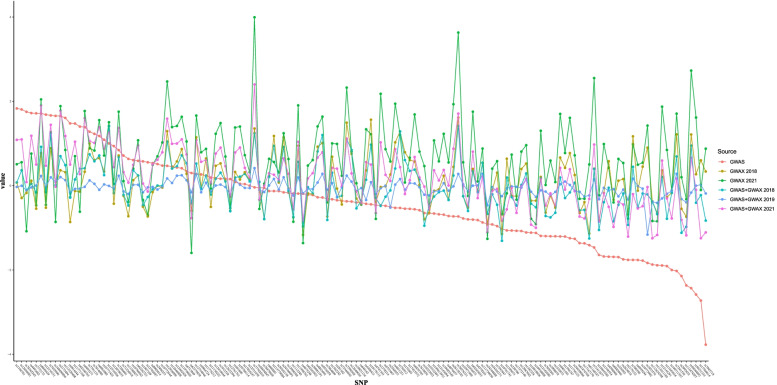
A combined plot visualizing the differences in effect sizes and directions across the six datasets using the 143 genetic variants. GWAS, genome-wide association studies; GWAX, GWAS by proxy; GWAS+GWAX, meta-analyses of GWAS and GWAX

These above MR analyses were based on different educational attainment genetic variants as the instrumental variables in AD GWAS, GWAX, and GWAS+GWAX datasets. We further conducted a secondary MR analysis using the same educational attainment genetic variants in each AD GWAS, GWAX, and GWAS+GWAX datasets. Finally, we selected 143 same educational attainment genetic variants available across all datasets by excluding rs268134 and rs28420834, as both are pleiotropic variants. Interestingly, the secondary MR analysis supported these above findings, as provided in Table 4. Figure 2 is a combined plot, which visualizes the differences in effect sizes and directions across the six datasets using the 143 genetic variants.

**Table 4:** MR analysis of the association between educational attainment and AD using the same educational attainment genetic variants

Dataset	Method	OR	95% CI	*P* value
GWAS	IVW	0.68	0.57–0.81	8.04E−04
	Weighted median	0.65	0.51–0.84	1.00E–03
	MR-Egger	0.39	0.19–0.82	1.20E−02
	MR-PRESSO	0.68	0.57–0.81	2.64E−05
GWAX 2018	IVW	1.09	1.00–1.19	5.00E−02
	Weighted median	1.01	0.89–1.16	8.67E−01
	MR-Egger	0.92	0.71–1.17	4.88E−01
	MR-PRESSO	1.09	1.00–1.19	5.17E−02
GWAX 2021	IVW	1.88	1.62–2.18	1.14E−16
	Weighted median	1.88	1.52–2.32	5.14E−09
	MR-Egger	1.88	1.04–3.39	3.60E−02
	MR-PRESSO	1.88	1.62–2.18	9.03E−14
GWAS+GWAX 2018	IVW	0.99	0.91–1.07	7.87E−01
	Weighted median	0.92	0.81–1.04	1.63E−01
	MR-Egger	0.95	0.74–1.22	7.05E−01
	MR-PRESSO	0.99	0.91–1.07	7.87E−01
GWAS+GWAX 2019	IVW	0.96	0.93–0.98	1.00E−03
	Weighted median	0.95	0.92–0.99	9.00E−03
	MR-Egger	0.91	0.81–1.01	7.03E−02
	MR-PRESSO	0.96	0.93–0.98	7.63E−04
GWAS+GWAX 2021	IVW	1.21	1.08–1.36	1.00E-03
	Weighted median	1.19	1.01–1.40	3.90E−02
	MR-Egger	1.03	0.65–1.65	8.90E−01
	MR-PRESSO	1.21	1.08–1.36	1.33E−03

The significance of the association between educational attainment and AD was at *P* < 0.05

*CI* confidence interval, *IVW*, inverse-variance weighted, *MR-PRESSO* Mendelian randomization pleiotropy residual sum and outlier, *GWAS* genome-wide association studies, *GWAX* GWAS by proxy, *GWAS+GWAX* meta-analyses of GWAS and GWAX

### Heterogeneity test and paired-samples *T* test

Using the 143 same educational attainment genetic variants, we tested the genetic heterogeneity of the Wald estimators in AD GWAS and GWAX datasets. Using GWAS vs. GWAS 2018 comparison, 47 (33%) and 29 (20%) of 143 genetic variants showed evidence of heterogeneity with *I*^2^ > 25% and *I*^2^ > 50%, respectively. GWAS vs. GWAX 2021 comparison indicated that 57 (40%) and 44 (30%) of 143 genetic variants showed heterogeneity with *I*^2^ > 25% and *I*^2^ > 50%. paired-samples *T* test showed that the average Wald estimator in GWAS (mean = − 0.39) was significantly smaller than the average Wald estimators in GWAX 2018 (mean = 0.13, and *P* = 2.17E−06) and GWAX 2021 (mean = 0.65, and *P* = 3.16E−14), respectively.

## Discussion

Until now, MR methods had been widely used to determine the causal association between AD and previously reported risk factors using the AD GWAS, GWAX, or GWAS+GWAX summary datasets [16–32]. However, the consistency of MR estimates from AD GWAS, GWAX, or GWAS+GWAX datasets currently remains unclear. Here, we first evaluated the causal association of a well-established risk factor educational attainment with the risk of AD using large-scale GWAS, GWAX, or GWAS+GWAX datasets [16–18, 33]. MR analysis in the AD GWAS dataset showed that high educational attainment could significantly reduce the risk of AD, which is consistent with recent findings [16–18, 33]. However, MR analysis indicated no significant causal association between educational attainment and AD in AD GWAX 2018 dataset, and even showed that high educational attainment increased the risk of AD in the AD GWAX 2021 dataset. Meanwhile, MR analysis in AD GWAS+GWAX datasets suggested the ambiguous findings about the causal association between educational attainment and AD. Hence, all these above findings indicated the inconsistency of MR estimates in AD GWAS, GWAX, and GWAS+GWAX datasets.

Interestingly, our findings are consistent with recent findings in AD genetic variants. Andrews and colleagues recently summarized and discussed 40 AD susceptibility loci with genome-wide significance, which were identified by at least one of the four studies [10], including GWAS 2013 [6], GWAS+GWAX 2018 [13], GWAS+GWAX 2019 [14], and GWAS 2019 [9]. They found that only 15 were replicated across all the four studies, and 9 were replicated in two or three studies at full genome-wide significance [10].

We further test the genetic heterogeneity of educational attainment genetic variants across the GWAS, GWAX, and GWAS+GWAX datasets. Interestingly, heterogeneity test indicated evidence of genetic heterogeneity across the GWAS, GWAS, and GWAS+GWAX datasets. Importantly, GWAS vs. GWAX 2021 comparison highlighted the largest number of genetic variants with heterogeneity. Hence, the genetic heterogeneity may have caused the opposite directions about the causal association between educational attainment and AD in clinically diagnosed AD and self-report proxy phenotype.

Our findings are consistent with recent hypothesis that the phenotypic heterogeneity may cause the genetic heterogeneity, and further reduce the statistical power for GWAX and GWAS+GWAX [10, 13]. It is known that the AD GWAX is based on the self-report AD proxy phenotype from UK Biobank participants [10, 13]. GWAX theoretically could increase the large-scale sample size into traditional AD GWAS, and further improve the statistical power [10, 13]. In fact, not all UK Biobank participants could discriminate AD from other dementia subtypes, and exactly reflect the clinically diagnosed AD status, considering the different presentations and genetic architectures [13]. Hence, the incorrect AD classification may reduce the statistical power to detect the true AD risk loci, and further influence the loci uncovered using GWAX and GWAS+GWAX [13]. This hypothesis may explain why only a small fraction of AD susceptibility loci could be replicated across the AD GWAS, GWAX, and GWAS+GWAX datasets. We think that this hypothesis and our findings from heterogeneity test may explain the inconsistency of MR estimates in AD GWAS, GWAX, and GWAS+GWAX datasets. Meanwhile, the biological factors and medical interventions may also have dramatically different effects on different people [43].

Our MR study may have several strengths. First, we selected one large-scale educational attainment GWAS dataset, and six large-scale AD GWAS, GWAX, or GWAS+GWAX datasets, which may provide ample power to detect the causal association between educational attainment and the risk of AD, as reported by recent MR studies [16–18, 33]. Importantly, all these participants are of European descent, which may further reduce the influence from population stratification. Third, we selected multiple MR methods and tested the pleiotropy. Hence, the MR assumptions did not seem to be violated. Fourth, educational attainment is well-established AD risk factor, as reported by recent MR studies [16–18, 33]. Hence, evaluating the impact of educational attainment on AD risk may exactly reflect the consistency of MR estimates from AD GWAS, GWAX, or GWAS+GWAX datasets.

## Limitations

Our MR study may have some limitations. First, we only selected one AD risk factor educational attainment to evaluate the consistency of MR estimates across AD GWAS, GWAX, or GWAS+GWAX datasets. In fact, several risk factors have been identified to be causally associated with AD risk [23, 26, 27, 44–52]. Hence, our findings should be further verified using other well-established AD risk factors. Second, the educational attainment of GWAS is based on the meta-analysis of SSGAC (293,723 individuals) and UK Biobank (111,349) [34]. Hence, the educational attainment GWAS dataset and GWAX, or GWAS+GWAX may have the overlapped individuals, and may not be independent. Hence, our MR findings from GWAX, and GWAS+GWAX should be further evaluated using independent datasets.

## Conclusions

In summary, our MR analysis highlighted the difference and genetic heterogeneity in clinically diagnosed AD and self-report proxy phenotype using large-scale AD GWAS, GWAX, and GWAS+GWAX summary datasets. Hence, the GWAX and GWAS+GWAX findings and MR findings from GWAX and GWAS+GWAX should be carefully interpreted and warrant further investigation using the AD GWAS dataset.

## Supplementary Information

**Additional file 1:** **Supplementary Table 1.** 162 independent SNPs that reached genome-wide significance (*P* < 5×10^-8^) in the pooled-sex *EduYears* meta-analysis of the discovery and replication samples (*N* =405,072). **Supplementary Table 2.** GWAS summary statistics of 159 educational attainment genetic variants in AD GWAS dataset. **Supplementary Table 3**. GWAS summary statistics of 147 educational attainment genetic variants in AD GWAX 2018 dataset. **Supplementary Table 4.** GWAS summary statistics of 159 educational attainment genetic variants in AD GWAX 2021 dataset. **Supplementary Table 5.** GWAS summary statistics of 147 educational attainment genetic variants in AD GWAS+GWAX 2018 dataset. **Supplementary Table 6.** GWAS summary statistics of 159 educational attainment genetic variants in AD GWAS+GWAX 2019 dataset. **Supplementary Table 7.** GWAS summary statistics of 159 educational attainment genetic variants in AD GWAS+GWAX 2021 dataset.

## Data Availability

All relevant data are within the paper. The authors confirm that all data underlying the findings are either fully available without restriction through consortia websites, or may be made available from consortia upon request. IGAP consortium data are available at http://web.pasteur-lille.fr/en/recherche/u744/igap/igap_download.php;https://www.niagads.org/datasets/ng00075.
